# P-1257. Development and Evaluation of a Vancomycin Pharmacokinetic Model Selection Algorithm: Further Refinement of Individualized Dosing in a Bayesian Software

**DOI:** 10.1093/ofid/ofaf695.1448

**Published:** 2026-01-11

**Authors:** Maria-Stephanie Hughes, Jon Faldasz, Ron J Keizer, Jasmine Hughes

**Affiliations:** InsightRX, Boston, MA; InsightRX, Boston, MA; InsightRX, Boston, MA; InsightRX, Boston, MA

## Abstract

**Background:**

Bayesian software programs have improved vancomycin target attainment by optimizing pharmacokinetic (PK) model selection. Here, we investigate how subgrouping patients by age, body mass index (BMI), sex and serum creatinine (sCr) can individualize model selection, and develop an algorithm for use at the bedside.

**Table 1:**
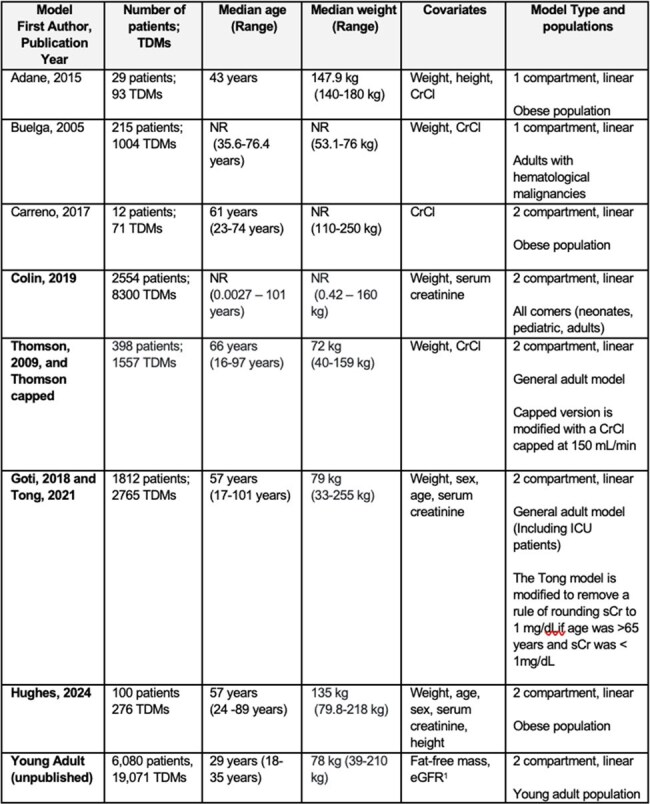
Summary of Models Considered for Implementation in the Model Selection Algorithm All models considered for the algorithm are summarized within the table. The bolded models are those that were included in the algorithm. The models by Adane and Buelga et al. were not included as they were 1 compartment models. The model by Carreno et al. was not considered due to practical experience showing instability in maximum a posteriori (MAP) Bayesian estimates, where small changes in covariates or sample timing led to large shifts in pharmacokinetic parameters.

**Table 2:**
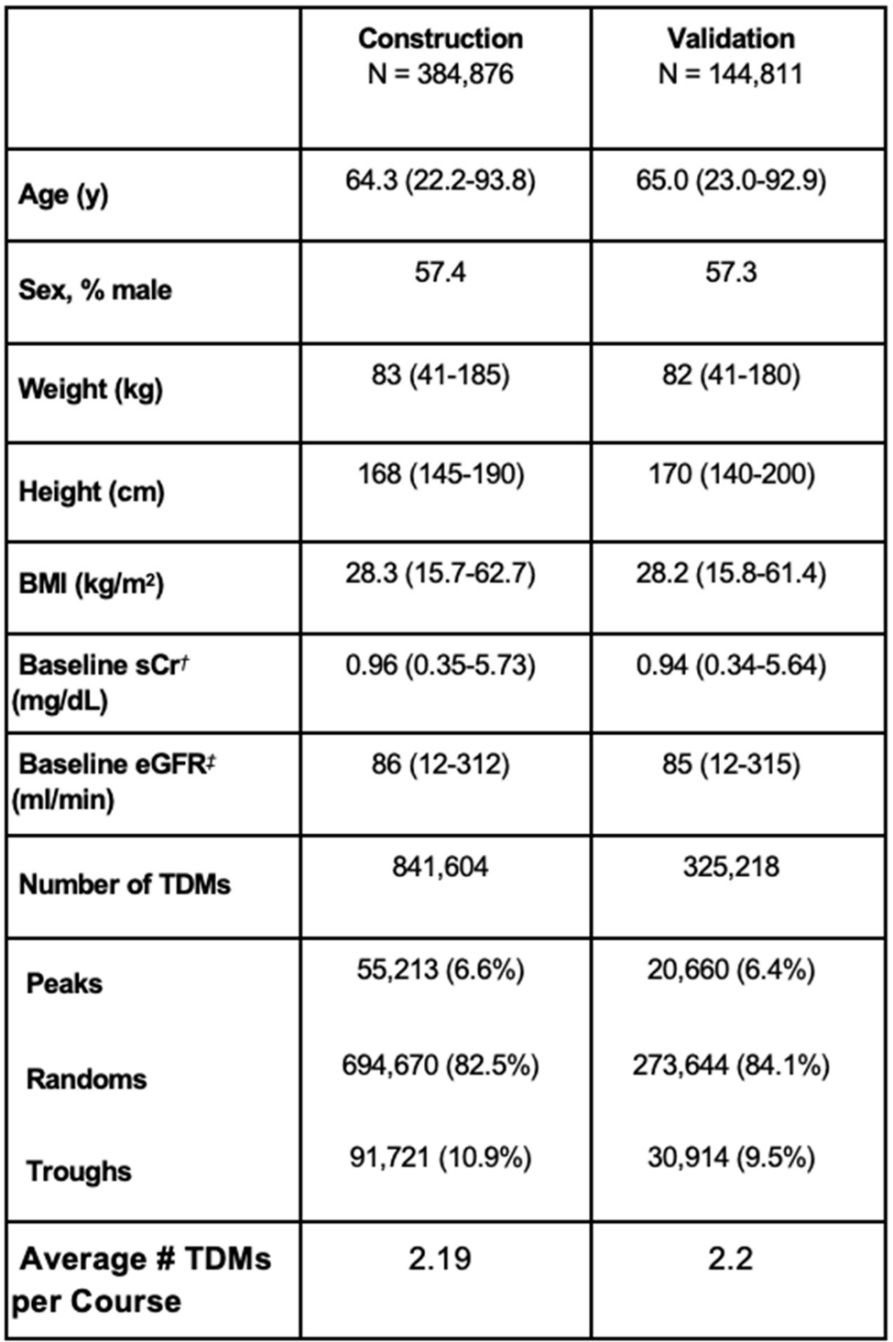
Patient Characteristics by Cohort sCr: serum creatinine. eGFR: estimated glomerular filtration rate. TDMs: Drug concentration levels. † Baseline sCr defined as first sCr value recorded per treatment course. ‡ Baseline eGFR was calculated using the Cockcroft-Gault equation, with total body weight, and baseline serum creatinine. Values indicate median (1st-99th percentiles) or counts (proportion) unless otherwise indicated.

**Methods:**

Vancomycin dosing data from patients treated between Jan 1, 2022 and Dec 31, 2023 were retrospectively collected from a Bayesian database. Patients were included if >18 years with ≥1 vancomycin level; excluded were those receiving < 2 doses or with levels drawn during infusion or before the first dose. Patients were grouped into 129 subpopulations based on age, sex, BMI, and sCr. Model predictiveness of 10 previously validated models (Table 1) were compared for each subpopulation. Predictiveness was measured by root mean square error (RMSE), mean percent error (MPE) and Accuracy both *a priori* and *a posteriori*. The algorithm was developed based on the following guiding principles: prioritizing *a priori* over *a posteriori*, overall model predictiveness assessed according to a novel equation (Figure 1), favoring overestimation of clearance over underestimation when bias magnitude is comparable, considering the model’s development population, preferring 2-compartment models, and accounting for practical factors. The final algorithm’s performance was compared to simple 2- or 3-model selection methods on a validation cohort of patients dosed from Jan 1st - June 16th, 2024.

**Figure 1.**
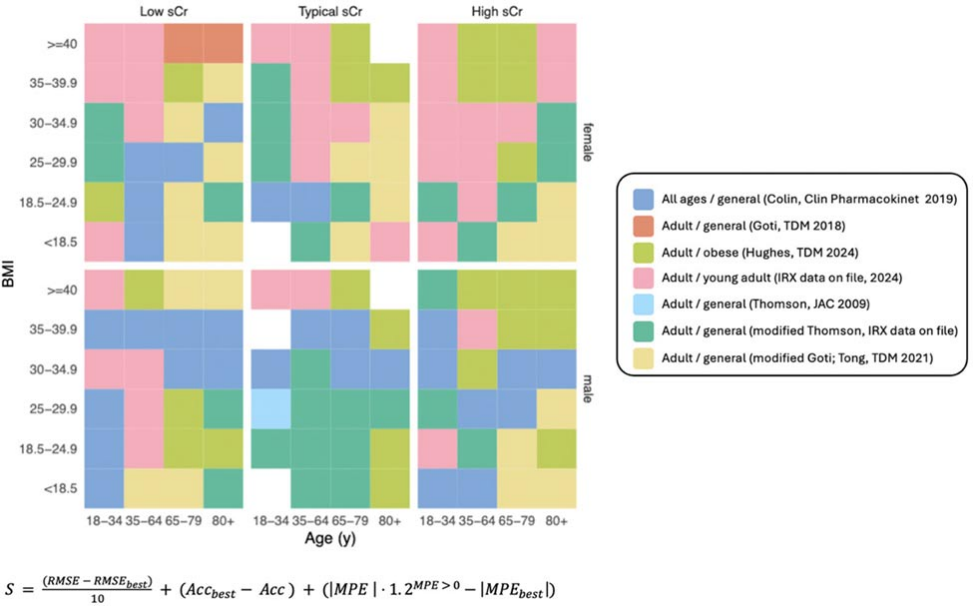
Model Selection by Age, BMI, sCr, and Sex Each color represents the model, as designated in the key, that is performing best for the associated subpopulation. sCr percentile bins are specific to each sex/BMI/age strata—0–25th, 25–75th, 75–100th percentiles; collapsed to 0–50th and 50–100th if bin sizes <200. Since the subpopulations with relatively few patients have been grouped into only two sCr bins (high and low sCr), and so the “Typical sCr” column contains some blank squares. The equation represents overall determination of predictiveness to select the “best” model.

**Figure 2.**
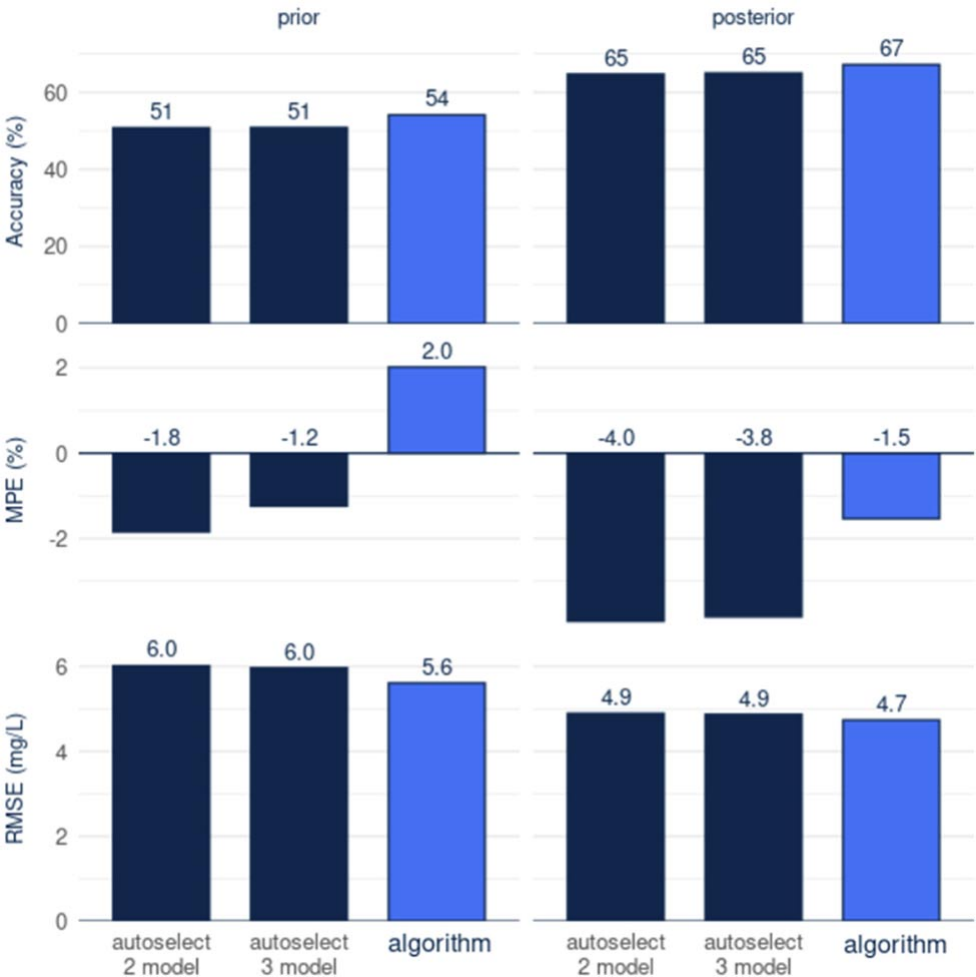
Predictiveness of the Algorithm Compared to a 2- and 3- Model Selection Method Accuracy, MPE as a measure of bias, and RMSE as a measure of precision are compared. Accuracy was as defined as the proportion of predictions falling within 20% or 2.5 mg/L of the observed data. The autoselect 2 model refers to utilizing the capped Thomson model and switching to Hughes model for patients of BMI ≥40 kg/m2; the autoselect 3 model refers to the same two model selection augmented with a selection of the Young Adult model for patients under the age of 35 years and BMI <40 kg/m2.

**Results:**

There was a total of 384,876 patients included in the algorithm construction population and 144,811 in the validation population (Table 2). Seven of the 10 models were considered and added to the algorithm, and each were found to be best performing for at least one of the 129 age-BMI-sCr-sex subpopulations (see Figure 1). The algorithm outperformed the 2- and 3- model selection methods in all predictive metrics *a posteriori*, and in all but one (bias) *a priori* (Figure 2).

**Conclusion:**

The model selection algorithm created in this study is a first of its kind and outperformed other model selection methods. It may improve vancomycin dosing decisions within Bayesian software, employing a transparent, decision tree–like algorithm and facilitating model selection across >100 subpopulations.

**Disclosures:**

Maria-Stephanie Hughes, PharmD, InsightRX: Employee of company|InsightRX: Stocks/Bonds (Private Company) Jon Faldasz, PharmD, BCPS, InsightRX: Employee Ron J. Keizer, PharmD, PhD, InsightRX: Ownership Interest|InsightRX: Stocks/Bonds (Private Company) Jasmine Hughes, PhD, InsightRX: Employee

